# Supplementary material for: The Inflammatory Response after Moderate Contusion Spinal Cord Injury: A Time Study
Source: Biology (Basel). 2022 Jun 20;11(6):939. doi: 10.3390/biology11060939 (PMC9220050; doi:10.3390/biology11060939)
Supplement: Supplementary file 1 [file biology-11-00939-s001.zip › biology-1627411-supplementary.pdf]

biology

MDPI

Article

The inflammatory response after moderate contusion spinal cord injury: A time study

Minna Christiansen Lund[1§], Ditte Gry Ellman[1§], Maiken Nissen[1], Pernille Sveistrup Nielsen[1], Pernille Vinther Nielsen[1], Carina Jørgensen[1], Ditte Caroline Andersen[2,3,4], Han Gao[5,6], Roberta Brambilla[1,7,8], Matilda Degn[9], Bettina H. Clausen[1,8], and Kate Lykke Lambertsen[1,8,10*]

[1] Department of Neurobiology Research, Institute of Molecular Medicine, University of Southern Denmark, Odense, Denmark; minnacl@hotmail.com (MCL); dellman@health.sdu.dk (DGE); maiken92@live.dk (MN); pernillesveistrup@hotmail.com (PSN); pvnielsen@health.sdu.dk (PVN); carinajoergensen@hotmail.com (CJ); rbrambilla@med.miami.edu (RB); bclausen@health.sdu.dk (BHC);; klambertsen@health.sdu.dk (KLL).

[2] Department of Clinical Research, University of Southern Denmark, 5000 Odense, Denmark; dandersen@health.sdu.dk (D.C.A.)

[3] Andersen Group, Department of Clinical Biochemistry, Odense University Hospital, 5000 Odense, Denmark

[4] Danish Center for Regenerative Medicine, Odense University Hospital, 5000 Odense, Denmark

[5] Department of Spine Surgery, The Third Affiliated Hospital of Sun Yat-sen University, 510630 Guangzhou, China; gaoh35@mail.sysu.edu.cn (HG)

[6] Guangdong Provincial Center for Engineering and Technology Research of Minimally Invasive Spine Surgery, 510630 Guangzhou, China.

[7] The Miami Project to Cure Paralysis, University of Miami Miller School of Medicine, 33136 Miami, USA.

[8] Brain Research Inter-Disciplinary Guided Excellence (BRIDGE), Department of Clinical Research, University of Southern Denmark, Odense, Denmark.

[9] Department of Pediatrics and Adolescent Medicine, Rigshospitalet, Copenhagen, Denmark; matildadegn@gmail.com.

[10] Department of Neurology, Odense University Hospital, Odense, Denmark.

§ Shared first authors

* Correspondence: e-mail: klambertsen@health.sdu.dk; phone. +4565503806.

Citation: Lund, M.C.; Ellman, D.G.; Nissen, M.; Nielsen, P.S.; Nielsen, P.V.; Jørgensen, C.; Andersen, D.C.; Gao, H.; Brambilla, R.; Degn, M.; et al. The Inflammatory Response after Moderate Contusion Spinal Cord Injury: A Time Study. *Biology* **2022**, *11*, 939. https://doi.org/10.3390/biology11060939

Academic Editor: Huaxin Sheng

Received: 21 February 2022
Accepted: 17 June 2022
Published: 20 June 2022

Publisher's Note: MDPI stays neutral with regard to jurisdictional claims in published maps and institutional affiliations.

Supplementary Table S1. Primers for real time RT-qPCR gene amplification. PCR efficiency repre-sents the average of two assays.

Gene	Accession No.	Size (bp)	Start-end	PCR (%)	eff.Pro-duct T_M	Ann. temp (T_a)
Tnf	NM_001278601.1	123	211-230<br>310-333	100.8	81.5°C	60°C
Tnfrsf1a	NM_011609.4	91	1024-1047<br>1014-1091	102.8	80.5°C	60°C
Tnfrsf1b	NM_011610.3	133	771-792<br>903-881	104.8	78.5°C	60°C
Il-1b	NM_008361.4	83	119-139<br>183-202	99.1	77°C	60°C
Il-6	NM_001314054.1	266	188-207<br>433-454	98.6	77.5°C	60°C
Il-10	NM_010548.2	424	213-237<br>615-637	96.45	81.5°C	61.5°C
Cxcl1	NM_008176.3	83	184-204<br>247-267	98.55	80°C	60°C
Itgam	NM_008401.2	86	3095-3114<br>3162-3181	101.2	79.5°C	55°C
Cx3cr1	NM_009987.4	85	79-98<br>145-164	99.4	77.5°C	55°C
Trem2	NM_031254.3	85	566-585<br>630-651	99.55	80.5°C	60°C
Arg1	NM_007482.3	87	332-351<br>400-419	100	81°C	60°C
P2ry12	NM_027571.4	85	139-159<br>205-224	98,75	81.5°C	60°C
Hprt1	NM_013556.2	80	107-126<br>166-187	101.8	84°C	60°C

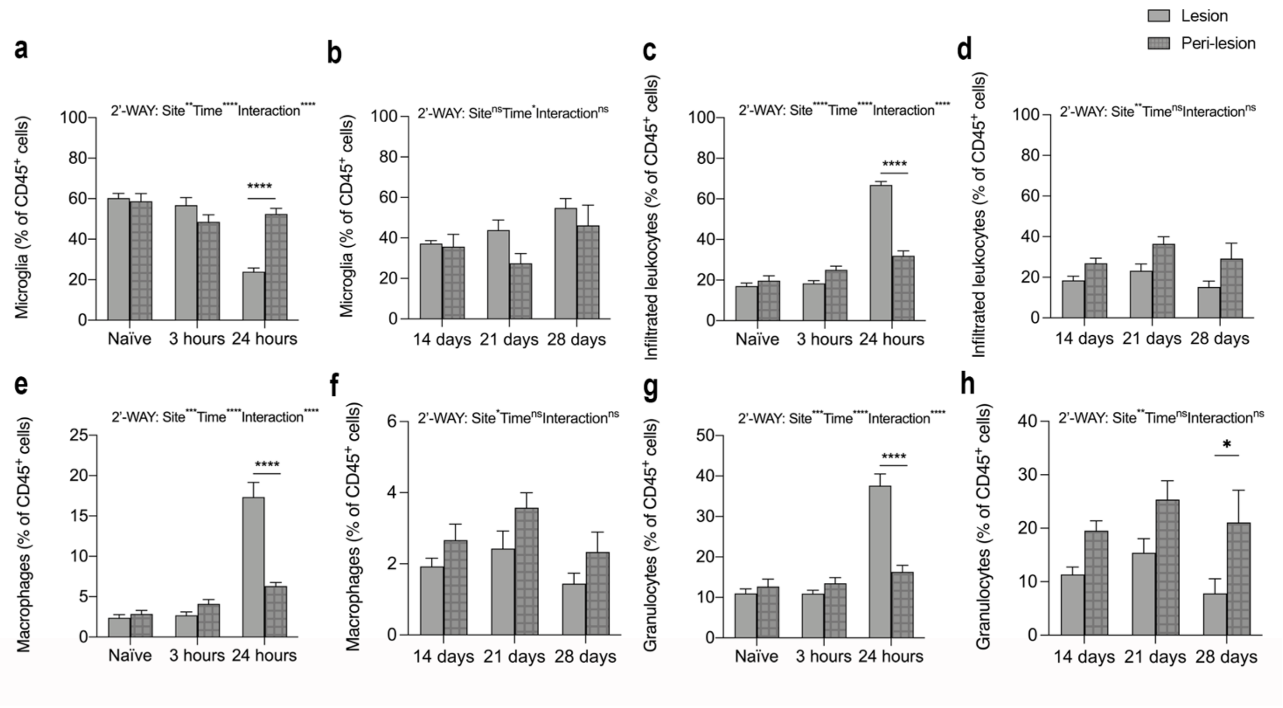

Supplementary Figure S1. Analysis of microglia/leukocyte populations after SCI. (**a**,**b**) Flow cy-tometric analysis of acute changes in microglial (a, Interaction: $F_{2,28}$=35.16, p<0.0001; Time: $F_{2,28}$=18.19, p<0.0001; Site: $F_{1,28}$=10.26, p=0.003) and chronic changes in microglial (b, Interaction: $F_{2,22}$=0.75, p=0.48; Time: $F_{2,22}$=3.62, p=0.04; Site: $F_{1,22}$=3.12, p=0.09) populations in the lesioned spinal cord. (**c**,**d**) Flow cytometric analysis of acute changes in leukocyte (c, Interaction: $F_{2,28}$=80.03, p<0.0001; Time: $F_{2,28}$=154.9, p<0.0001; Site: $F_{1,28}$=32.13, p<0.0001) and chronic changes in leukocyte (d, Interaction: $F_{2,22}$=0.26, p=0.77; Time: $F_{2,22}$=1.99, p=0.16; Site: $F_{1,22}$=11.65, p=0.003) cell populations in the lesioned spinal cord. (**e-h**) Infiltrated leukocytes were sub-gated into macrophages (e, Interac-tion: $F_{2,28}$=31.60, p<0.0001; Time: $F_{2,28}$=66.72, p<0.0001; Site: $F_{1,28}$=17.75, p=0.0002 and f, Interaction: $F_{2,22}$=0.11, p=0.89; Time: $F_{2,22}$=3.18, p=0.06; Site: $F_{1,22}$=6.69, p=0.02) and granulocytes (g, Interaction: $F_{2,28}$=39.45, p<0.0001; Time: $F_{2,28}$=37.67, p<0.0001; Site: $F_{1,28}$=20.19, p=0.0001 and h, Interaction: $F_{2,22}$=0.28, p=0.76; Time: $F_{2,22}$=1.64, p=0.22; Site: $F_{1,22}$=13.72, p=0.001). Open bars; lesion, checkered bars; peri-lesion. Results are presented as mean ± SEM, n=5-11/group, ****p<0.0001.
